# Methods of, and Materials for, Filling Teeth

**Published:** 1876-09

**Authors:** 


### ARTICLE III.

### *Methods of, and Materials for, Filling Teeth.*

The meeting was called to order by the President, Dr. J S. Latimer, and the minutes of the last meeting were read by the Secretary, and approved. After the usual business of the Society the subject of the evening was announced by the President as open for discussion.

Dr. A. C. Hawes said: I like the material of tin for filling teeth better than almost anything I have ever used. It seems to keep the teeth from decaying, as the acids of the mouth have seemingly a greater affinity for the tin than the teeth. I have used it a great deal, both for temporary and permanent fillings.

Dr. Chittenden: I do not know as I ought to say that I entirely endorse Dr. Hawes' theory with regard to galvanic action. We all know that galvanic action acts most rapidly near the point of the poles. Tin is one of the greatest preservers of the teeth known. I speak a little from experience. When my wife was thirteen years old, Dr. Tripp filled her teeth with tin, and ten years ago, when connected with the Academy of Science, I proposed this material for children's teeth. Another reason is that you can put it in position where you cannot possibly put gold. I can fill a tooth with tin foil so as to preserve the mere frame of a tooth, and children's teeth, to my mind, better than any other substance.

Dr. Wm. Allen: The subject is so large I hardly know where to begin or where to leave off. In my practice of filling children's teeth I have used very little tin. For teeth of a good character I use amalgam. I think anybody can fill teeth for children with amalgam better than they can with anything else, and I find that it lasts well. It can be put in with more certainty of being put in thoroughly than anything else, and I generally use it. It is cheaper for the patient, and answers the purpose, with me, perfectly. For teeth of a poor character it does not answer so well; for such teeth I use gold.

Dr. C. E. Latimer: For most children's teeth I think the best possible filling is amalgam. I use the best article I can get, and the effect has been very favorable. The alloy is made soft first, and the excess of mercury removed by squeezing in chamois. I advise washing with soda bicarbonate. If the surface of a metal is exposed to the air it oxidizes rapidly. If you have both sides exposed it oxidizes on both sides. If the metal is filled up you have a good many surfaces exposed, on which oxide remains in the filling unless washed out. By keeping the mercury under alcohol in suitable apparatus, there is very little oxidation.

Dr. Todd: With regard to children's teeth I would say that in my practice I have found that in most cases Hill's stopping answers a very good purpose indeed.

Dr. Atkinson: If I must say what I have to say, I think that the operator has more to do with the filling than any of the materials. I think that a good operator will be successful with almost any material that he has on hand, and when gentlemen talk about chemical results beforehand, they have too much faith for me, and when we talk about having everything ready, and about a result that we know no more about than we can deduce from our own unaided sight, I hold we ought to be careful about pronouncing anything as to the success or failure of fillings. It does not come in the art of human sharpness of insight to determine whether the filling material is in contact with the cavity or not. Any cavity that is perfectly impervious to moisture and to air around each margin, may be filled with any material not soluble in the fluids of the mouth. I have used all the substances that have been referred to to-night, and at one time I would run to one by preference, and at another time by preference to another, and standing as I have stood, I have been forced to make experiments and trials of things that I had not much confidence in simply because I have been continually asked to give an opinion, and I wanted to have an intelligent opinion to give. But when we speak about what is the best material for filling teeth, and the best mode in which to apply it, you must make a specific statement of what the case is. Now I am very much in favor of oxychloride of zinc. Properly prepared, and put in just at the right time, it makes a most excellent filling, if you bring the oxychloride of zinc, in a plastic state, against the ends of the dentine, so that the cavity is kept open by the fluid. It is not proper that the oxychloride of zinc should be beaten into the cavity. If mixed with any other substance, so as to make it plastic enough to take its place around the exposed pulp and leave margin enough in the dentine, then you can put it in with perfect safety. If the margin of the tooth is impervious, the tooth is safe. It is the margin we have to look at particularly.

There seems to be a misapprehension about the law of chemical action, of which we all acknowledge most of the decayed teeth coming through our hands, as the result. There must always be a current, where chemical action takes place, going from the positive to the negative pole—or, in other words, if there be two substances, the one electro-positive and the other electro-negative, connected, a current is set up between the two. We are in the habit of saying that there is an electro-negative condition of this tooth, and we desire to have it electro-positive. Now, the two substances of gold and dentine are so exactly alike that they are almost always in balance.

When Mr. Williams will bring me a case of decolorization of pure gold that has been properly cleansed, in the mouth of a "good housekeeper," then I will consider that question. I have seen, from the same sheet, teeth filled that would turn, and the same gold, No. 5 foil, make a good filling, without any change at all. I think it is caused by carelessness on the part of the person whose teeth are filled.

Dr. Burroughs: This takes me back some 40 years from the commencement of my experience. The saying is, "History repeats itself." Humanity repeats itself, and I find that the material practice of dentistry has repeated itself. I used to be, in my earlier days, very fond of using tin as a filling, until some of the older ones began to blackguard me for using it. The principle of using the tin is right, but all depends in a great measure upon the operator, and the manner in which the operation is performed, and conditions requiring special material to be used. The packing process, many years ago, was used with great success. Now I find that new cements have come up—the oxychloride, a comparatively new thing, and still used in a great measure, but the old-fashioned cement is nothing more or less than an improvement on tin, and has been used to great advantage. I am in the habit of using all material, oxychloride, tin, gold, cement, or anything that comes along if I find the peculiar condition meets the case required. I do not give any preference to any particular one, sometimes filling with tin. It is a very excellent plan which I adopted many years ago, and I still continue to use it. I cannot say as much for my friends, brothers Latimer and Allen.

In the way of amalgam, I have not had as good results, probably owing to the conditions of the cases and the manipulation. I shall next hear that the introduction of arsenic and creosote, in destroying the nerve, which was very popular at one time, and has been used very extensively, and has now probably died of old age, will repeat itself.

Dr. Stockton: "The manner of filling teeth and the materials used in the operation" is a subject in which we all are very deeply interested. I simply wish to give two or three hints. In filling the approximal cavities in the bicuspids and molars, when both the anterior and posterior surfaces are decayed, my process has beeen to fill the anterior cavity of the posterior tooth first, and without finishing off the filling and inserting a matrix for the filling of the posterior cavity in the anterior tooth, as many do, I continue to build my gold right over into it, thus making it really one solid filling, and when done all you have to do is to seperate with a fine saw, or file and finish off in the usual manner; and thus you have the original form of the teeth, and it is much the easier way to me.

Another hint: do not wash your amalgam. I had formerly taken great pains to do so, and found, notwithstanding all my care, that it shrank from the walls of the cavity. I have lately had under my observation a large number of amalgam fillings not washed, and they are excellent in every respect except in color; they have saved the teeth in which they are inserted for several years, and will continue to do so for several years to come, I have not the least doubt. Since this experience, and also the careful attention given the subject by Dr. Bogue, I have ceased to wash my amalgam, and think, from careful examination of the results, I shall continue not to wash it. Use the rubber dam, pack the amalgam with the same care as gold, and when you have finished and burnished it varnish with sandrach, and you will have good results.

In regard to the gutta-percha, it is one of the best pain obtunders to be found; insert it in the tooth and let it remain for three or four weeks, and you can generally finish the operation at that time with little or no inconvenience to your patient. It makes a good permanent filling on the labial surfaces, where there is no force of mastication upon it.

Again, in filling teeth—as Dr. Atkinson has so well just said—it all depends upon the operator. The work *must* be thoroughly done, and here is where I can ride on my hobby—the motor. With it you can do your work better and save yourself so much labor. I can stand at my chair from morning to night, day in and day out, and with its help work is a pleasure. If one does his work thoroughly, it is not so much the materials used, after all, whether it be gold, tin, amalgam or anything else. There ought to be no such thing as a poor dentist.

Dr. Miller: I am like many others. I use gold, tin, amalgam, and all the rest of the materials. In some teeth I think it is better to use amalgam, and sometimes I think it is best to use amalgam even if tin would answer the purpose as well or better, if you could get it in. The other day I was at work, and I could not get along as well putting in tin as amalgam, and so I used gold. I seldom use oxychloride in children's teeth, from the fact that I think it should be kept dry for so long a time, or else it will waste away.

Dr. F. M. Odell: One gentleman tells us how, before he had a motor, he did not cut out his cracks. Now before I

had an engine of any kind, except *ingenuity*, I did cut out the cracks. It did not add much to the pocket, it is true, because other people who did not cut them out could do it three or four times as quickly as I could; but I have some little monuments standing of good work, done at a time when, I confess now, I did not know how to fill teeth.

In regard to tin. I should think I have put in a dozen fillings in the course of thirteen years; can pack gold easier and better. Where I cannot use gold I have never been ashamed to say I use amalgam. Before Dr. Bogue issued his ukase I washed it; but since then I have never done so, and I have yet to see a difference in the cases where I washed it and where I did not. I have some amalgam plugs, of which I am just as proud as if they were gold. I have done some work with Hill's stopping. I put in one plug for my wife and it stood there eight years, right in the crown of a molar tooth. Of course it did not come into much wear, you would say, but the plug remained after the tooth had decayed away from it on every side. The tooth was lost because, after this first experience in dentistry, she would never let me touch that part of her again.

Dr. Jarvis: I have been summing up my experiences during the past few months more carefully than ever before and extending through a number of years. What is to sum up? How can we determine what we have attained unto? One process by which I have endeavored to accomplish this has been to save the deciduous teeth. I have a bottle containing scores of them. One collection I have contains the entire number of teeth of my two children. In doing this I have a great variety, exhibiting different stages of decay and different kinds of filling, and fillings showing different results. To explain the matter more clearly take the teeth of my own children. Some of the deciduous teeth were filled with tin. I always liked tin very well because, in the winter of 1848-9, as I was preparing the gold to fill some teeth for an old lady, says she, "What are you going to do with that?" "I am going to put it in your teeth." "No,"

says she, "I don't want any thing of that kind in my teeth," and then she showed me two tin fillings that had been there for 32 years. Sometimes it would oxidize. More frequently it would wear away. Gutta-percha stopping has resulted about the same with me in a great majority of cases, and I have found it to rot—positively rot—exhibiting a perfectly disintegrated surface, ready to move away almost at the slightest touch. After all the pains I could take in using it, in a great majority of cases it has disappointed me.

In regard to amalgam in filling deciduous teeth, I have found it to *generally* oxidize. I find that the great difficulty is oxidation. In those that I have collected you can see a tendency in that direction. Others of these specimens I filled with gold, and I have had better results. There is a difficulty in getting in there, perhaps; but I wish to say this much in regard to gold: I can scarce conceive of a case where I cannot succeed better with gold than with anything else. I believe that gold can be used in all cases. As far as the accessibility of the cavity is concerned, we *should* make it accessible, and when we have access to the position we can do what we please with the gold. I collected quite a quantity of plugs (I do not like that word "plugs," it doesn't sound quite right, but it may be the best thing after all,) that had been used as gold fillings. I went to a society once with a lot of these fillings, for the purpose of handing them around, and I have never seen them since. I am inclined to believe that I pulled them out of my pocket and lost them, but a number of persons examined them in my office before the time to which I referred. It was easy to see the causes of failure in these fillings. By these two methods—examining teeth and fillings—I have been able to arrive at conclusions more satisfactory to myself than looking into the patient's mouth.

I used to wash amalgam. I was told that I could not wash the black oxide all away. That is not my experience. It grows less and less—satisfactorily. Then, the most of that is probably caused by mercury, for the mercury oxides very rapidly when exposed to the atmosphere. That wants to be washed away, and the material brought into a mass as soon as possible. I have often seen these amalgam fillings when there was no oxide upon the surface exposed.

Dr. Bogue: The idea of washing I cannot approve of for two or three reasons. In the first place, it prevents the perfect consolidation of the filling afterwards, as the water cannot be perfectly expressed. In the next place, mercury will continue to oxidize, and to yield its black oxide, just so long as it is exposed to the atmosphere. The sooner, therefore, it can be combined with the alloy of which it is henceforth to be a part, the better. It is possible to put in an amalgam filling without washing, with less mercury than is ever used when it is washed. The reason for using little mercury are, to make a tight filling, to make a filling that will keep its color better, and most important of all, to make a filling that sets quickly, giving the mass as little time as possible to be acted upon by centripetal force, which tends to draw it together into the shape of a drop or sphere, which movement constitutes the balling or drawing up in the middle and away from the edges of the cavity so much complained of.

Dr. Francis: The material which answers the best purpose in one case, may not in another. There are a good many things to be taken into consideration. The size and shape of the cavity, and the position of the cavity. I think that in a majority of cases gold is the best material that has yet been discovered, although there are a good many cases where gold will not prevent teeth from decay.

It has been said that everything depends upon the manner in which it is filled. Certainly a good deal does depend upon how the operation is performed. Sometimes it is impossible to use gold as a filling. Just so with amalgam. I have known amalgam to not last five years. And just so with gold. So more depends upon the nature of the teeth, really, or quite as much, as the filling. I saw a filling once after it had been in a tooth for twenty years, and no decay; but the filling projected, and had done so for a long time. I considered gold in a majority of cases the best filling, but I should hardly think of using gold in children's teeth.

Dr. Stockton: In regard to the manner of filling teeth, which has been spoken of here to-night. In filling the under surface, for instance, of the two lower molars, my process has been to fill the posterior tooth first, and then finishing by making it really all one filling. When they were both decayed, I made my filling all into one, and went right along, and when done you can go right between them and separate the filling very quickly, and it is much easier than any other way I have found.

Dr. Odell. I would like to say a word in relation to Dr. Bogue's suggestion that tin foil rubbed upon the surface of the amalgam may be the thing to preserve the color; I have always burnished a portion of gold or tin foils upon the surface of my amalgam plugs. I see some of mine which turn slightly greenish sometimes, but which if brushed with pumice will always keep bright; I usually use gold for this purpose.

It was unanimously resolved that a vote of thanks be tendered to the gentlemen who so kindly cliniced for us to day.

Subject for next meeting—"Manner of Inserting Fillings in the Teeth and the Principles Involved in the Operation."

Dr. Stockton consented to furnish a paper on the subject. —*Proceedings First District Dental Society of N. Y.—Dental Miscellany.*

